# Bevacizumab and wound-healing complications: a systematic review and meta-analysis of randomized controlled trials

**DOI:** 10.18632/oncotarget.12666

**Published:** 2016-10-14

**Authors:** Hongliang Zhang, Zhenguang Huang, Xiaoqin Zou, Taotao Liu

**Affiliations:** ^1^ Pharmacy Department, The First Affiliated Hospital of Guangxi Medical University, Nanning, 530021, China

**Keywords:** bevacizumab, wound-healing complications, systematic review, meta-analysis

## Abstract

A meta-analysis was conducted to estimate the risk of wound-healing complications in patients who treated with neoadjuvant-adjuvant bevacizumab in various oncological indications. We searched PUBMED, EMBASE and the Cochrane Library through June 2016 to identify randomized controlled trials of bevacizumab and wound-healing complications. Seven RCTs studies involving 5,147 participants were included in the analysis. Compared with routine therapy, bevacizumab increased the incidence of wound-healing complications for various cancers. The pooled estimate of odds ratio (OR) was 2.32, and the 95 % confidence intervals (CI) was 1.43 to 3.75. (*P* < 0.001). Subgroup analyses revealed the similar result in colon carcinoma patients. In conclusion, bevacizumab increases the incidence of wound-healing complications for cancers especially for colon neoplasms patients. However, the adverse effect is not appeared in breast cancer, metastatic renal cell carcinoma, non-small-cell lung cancer and gastro-oesophageal adenocarcinoma. Due to the findings relying chiefly on data from single or two studies, hence, further research is required to assess the wound-healing complications risk of bevacizumab in each oncological indication.

## INTRODUCTION

As an anti-VEGF monoclonal antibody, bevacizumab (Avastin) was approved for treatment of metastatic colorectal cancer (mCRC), metastatic breast cancer(MBC), metastatic non-small cell lung cancer (NSCLC), metastatic renal cell cancer (RCC), and glioblastoma multiforme (GBM) by the Food and Drug Administration [1]. For metastatic breast cancer, bevacizumab has not shown a benefit, in terms of delay in the growth of tumors, that would justify its serious and potentially life-threatening risks. Nor is there evidence that use of Avastin will either help women with breast cancer live longer or improve their quality of life. FDA removed the indication of metastatic breast cancer from bevacizumab's product labeling since November, 2011 [2].

Although bevacizumab has clearly demonstrated antitumor efficacy, its mechanism of action is not fully understood. According to the current point of view, the mechanism of bevacizumab for tumor growth and progression included (1) inhibiting the growth of new vessels (GBM) [3], (2) regression of newly formed vasculature (mCRC) [4], (3) altering vascular function and tumor blood flow (normalization of the vasculature to transiently improve the delivery of and increase the efficacy of cytotoxic agents) (RCC) [5], and (4) direct effects on tumor cells (MBC) [6].

Owing to the widespread use of the bevacizumab, understand its particular toxicity profile will become increasingly important. As an antiangiogenic agent by the inhibition of VEGF, bevacizumab also mediates many normal physiological processes, leading to multiple adverse reactions including hypertension, hemorrhage, gastrointestinal perforation, arterial thromboembolism, and hypersensitivity reactions [7]. Particularly, angiogenesis is also crucial for proper wound repair, so bevacizumab also result in an increased risk of impaired wound healing [1, 8], which are needed to pay attention in the perioperative care of patients receiving such treatment.

Therefore, knowledge of the characteristic of bevacizumab-induced wound-healing complications is increasingly crucial to guide treatment and optimal evidence-based management recommendations.

So far, many of the large-scale clinical studies conducted to test the efficacy and safety of bevacizumab in its various oncological indications have definitely reported wound-healing complications [9–16]. However, these studies have conveyed conflicting results. Apart from one systematic review including one RCT study and other types of studies involving case control, cohort and case series [17], there remains no high-quality meta-analysis concerning the probability of wound-healing complications resulting from bevacizumab use.

Thus, to provide the latest and most convincing evidence, we conducted this meta-analysis to estimate the risk of wound-healing complications in patients who treated with neoadjuvant-adjuvant bevacizumab in various oncological indications.

## RESULTS

### Study identification and selection

**Figure 1:**
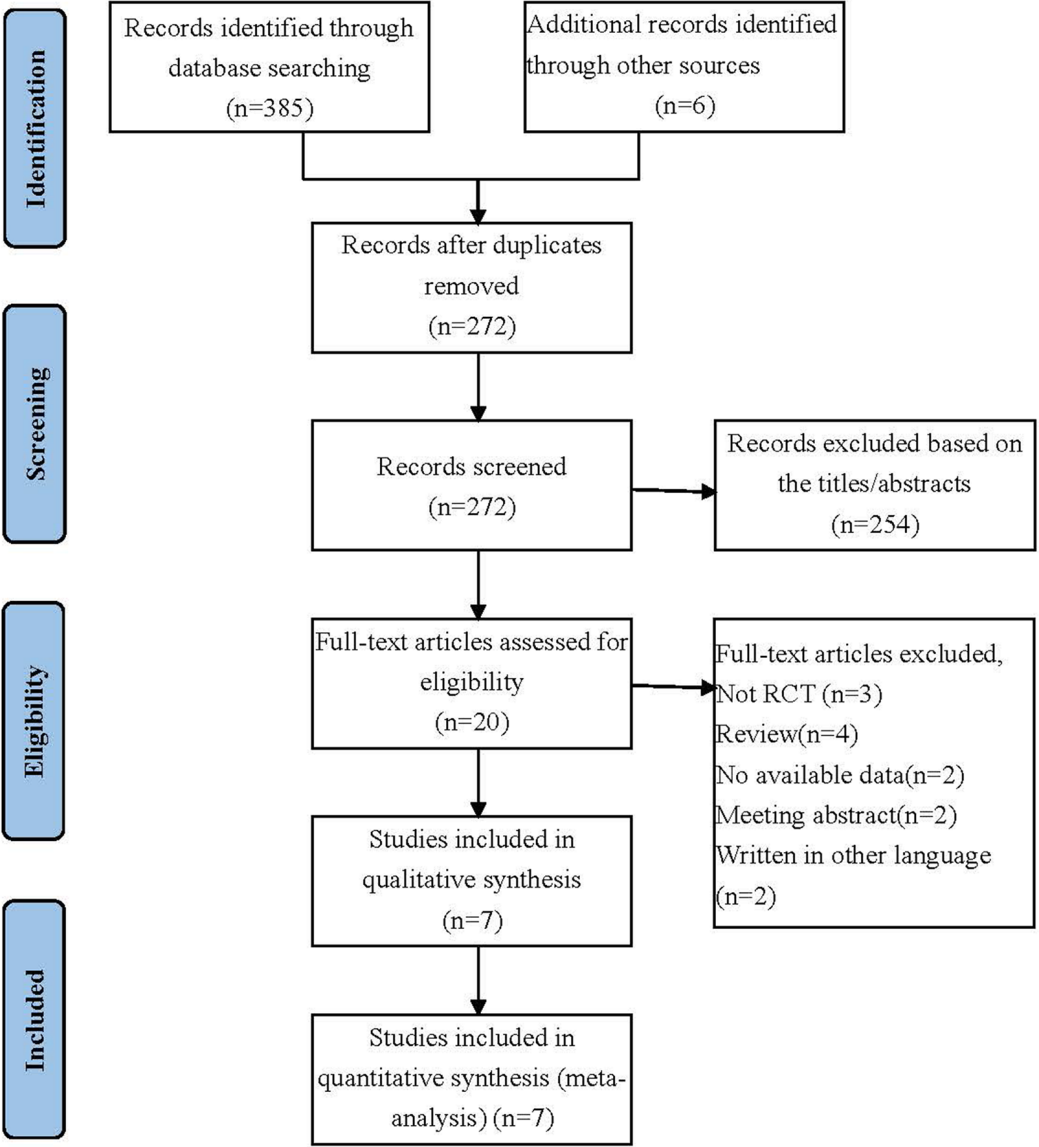
Selection process for the studies included in the meta-analysis

A total of 391 records were retrieved from the initial database search. After removing duplicate articles, 272 records were eligible. Based on the inclusion and exclusion criteria, 254 articles were excluded after a simple reading of the titles and abstracts of the articles. The remaining 20 full-text articles were assessed for eligibility. Furthermore, not RCT, review, no available data, meeting abstract, written in other language were excluded. Finally, a total of 7 RCTs studies were included in the meta-analysis [9, 11–16]. The selection process is shown in Figure 1.

### Study characteristics

The characteristics of the included studies are summarized in Table 1. Seven RCTs studies involving 5,147 participants were included in the analysis. These studies were published from 2007 to 2014.The number of participants in the studies ranged from 185 to 2,647. Among the included trials, 2 out of the 7 RCTs compared the efficacy and safety with or without bevacizumab for metastatic colorectal cancer [12, 13]. Two RCTs compared the outcome of bevacizumab and chemotherapy for breast cancer [15, 16]. The remaining 3 trials compared the roles of bevacizumab in metastatic renal cell carcinoma, non-small-cell lung cancer, gastro-oesophageal adenocarcinoma, respectively [9, 11, 14].

**Table 1:** The characteristics of included RCTs

Studies	Sample size		Interventions		Treatment period	Wound-Healing measures indicators	Indication
	Treatment group	Controlgroup	Treatment group	Control group			
Escudier 2007 [5]	327	322	Bevacizumab (10 mg/kg) + interferon alfa	Placebo + interferon alfa	every 2 weeks until disease progression	Wound healing complications	metastatic renal cell carcinoma
Allegra 2009 [9]	1321	1326	Bevacizumab (5 mg/kg) + mFOLFOX6	mFOLFOX6	every 2 weeks for a year	Wound complications	Colon Cancer
Miles 2010 [11]	495	241	bevacizumab (7.5/15 mg/kg) + docetaxel	placebo + docetaxel	every 3 weeks until disease progression	Wound-healing complication	Breast Cancer
Blumenschein 2011 [10]	63	123	bevacizumab (15mg/kg) + paclitaxel + carboplatin	motesanib + paclitaxel+carboplatin	every 3 weeks until disease progression	Impaired wound healing	non-small-cell lung cancer
Guan 2011 [8]	139	64	Bevacizumab (5 mg/kg) + mIFL	mIFL	every two weeks until disease progression	wound healing complications	metastatic colorectal cancer
Okines 2013 [7]	99	101	bevacizumab (7.5 mg/kg) + ECX	ECX	3-weekly	wound complication	gastro-oesophageal adenocarcinoma
Gerber 2014 [12]	394	349	bevacizumab (15 mg/kg) + epirubicin/cyclophosphamide + docetaxel	epirubicin/cyclophosphamide+ docetaxel	every 3 weeks for 8 cycles	Delayed wound healing	Breast Cancer

### Risk of bias assessment

**Figure 2:**
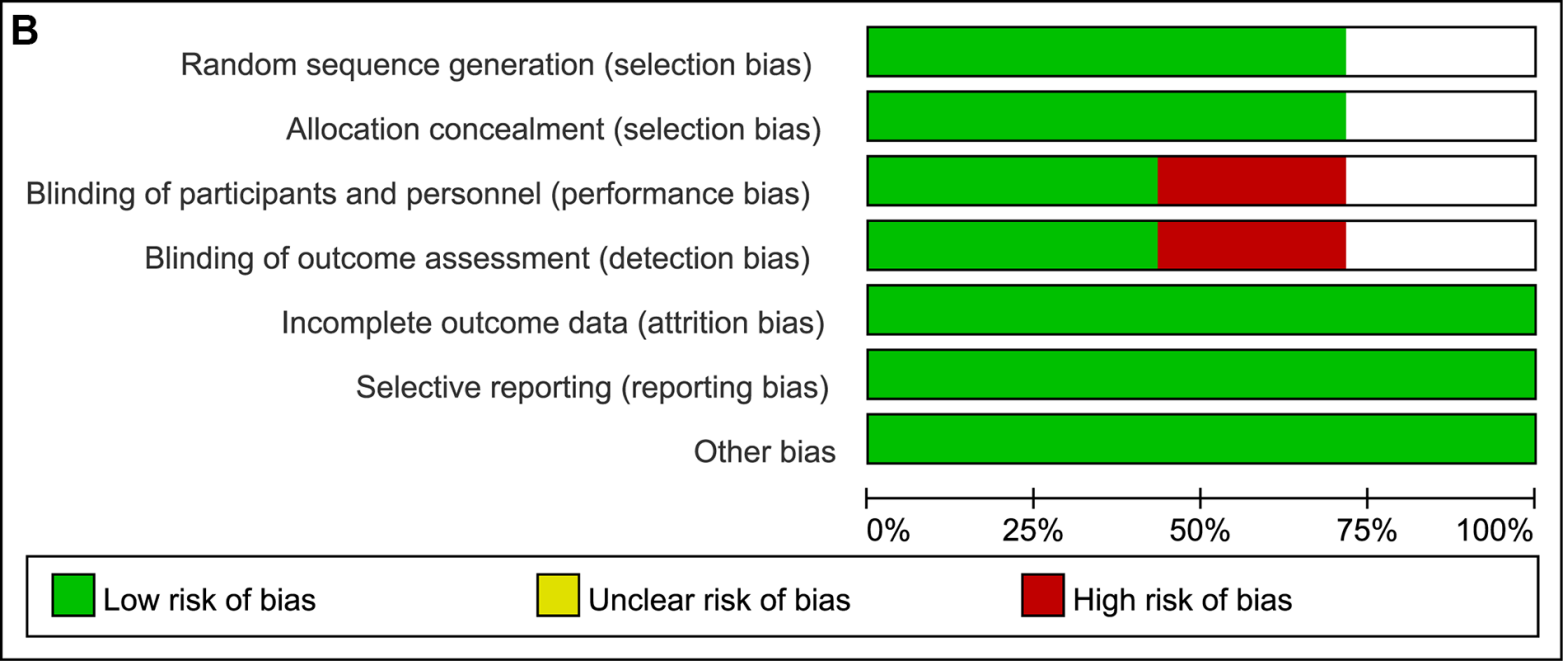

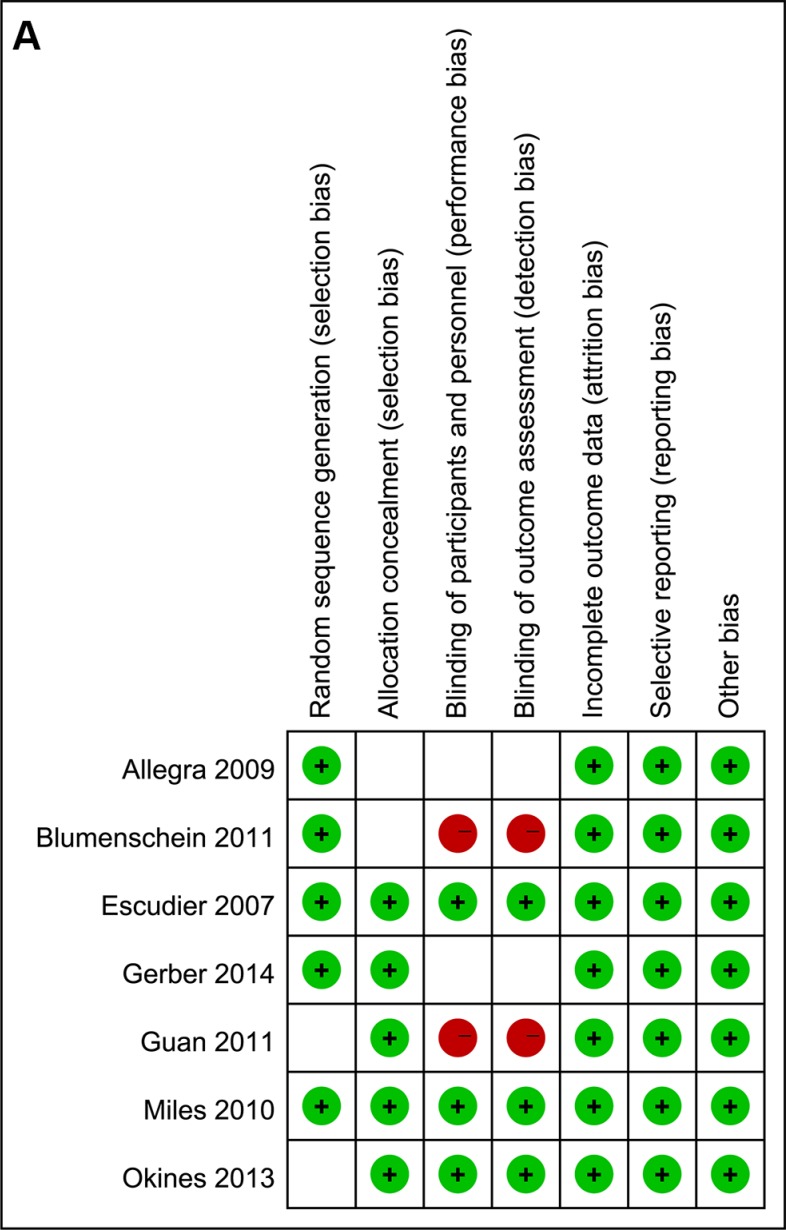
(**A**) Risk of bias summary; (**B**) Risk of bias graph

Theoutcomes of risk of bias are summarized in Figure 2A and Figure 2B. Among the studies included in the analysis, 5 described the randomization processes that they had employed [9, 13–16]. The allocation sequence concealment was not reported in the studies by Allegra [13] and Blumenschein [14]. Apart from 3 studies [9, 11, 15], blinding of outcome assessments was unclear or seldom reported in other trials. Besides, all studies did not have selective reporting bias.

### Primary outcome

**Figure 3:**
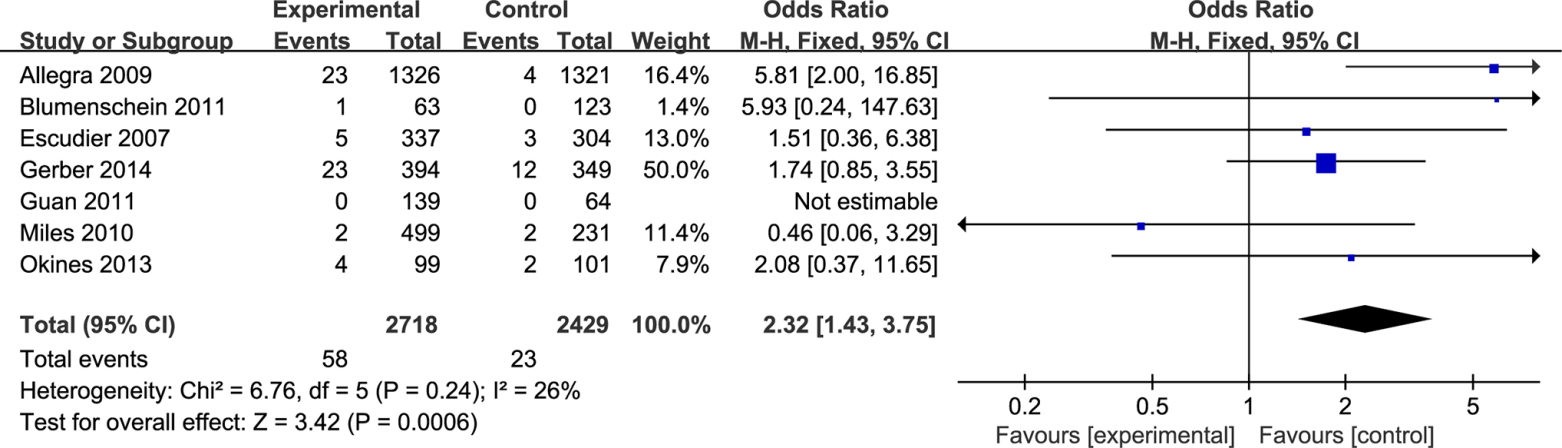
Incidence of wound-healing complications in bevacizumab versus control group

Seven studies totaling 5,147 patients provided data on wound-healing complications. Compared with routine therapy, bevacizumab increased the incidence of wound-healing complications for various cancers. The test for heterogeneity of 7 studies demonstrated no heterogeneity (*P* = 0.13; *I*^2^ = 26%), and the fixed effect model was performed. Based on our analysis, the pooled estimate of odds ratio (OR) was 2.32, and the 95% confidence intervals (CI) was 1.43 to 3.75. (*P* < 0.001). The result suggested that bevacizumab might be increased risk of wound-healing complications in patients with various oncological indications (Figure 3).

**Figure 4:**
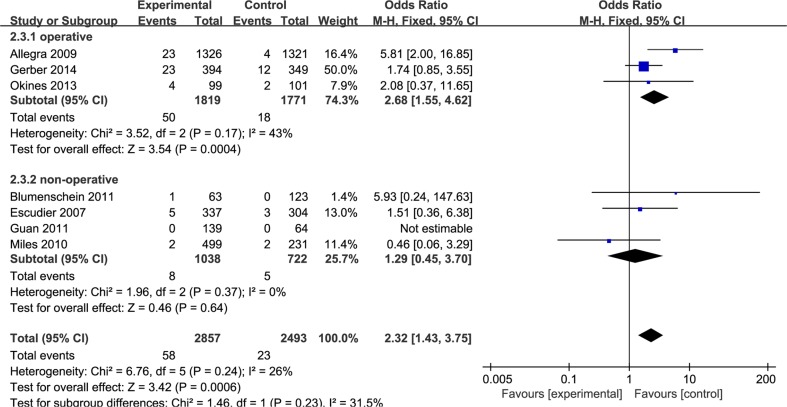
Subgroup analyses with different oncological indications

It's necessary to conduct subgroup analyses, due to bevacizumab was treated with diverse oncological indications. For colon cancer, two of the RCTs enrolled 2850 participants with 1465 patients assigned to the experiment group and the other 1385 patients assigned to the control group. Based on our analysis, the pooled estimate of odds ratio (OR)was 5.81, and the 95% confidence intervals (CI) was 2.00 to 16.85. (*P* < 0.05). This revealed that bevacizumab might be increased risk of wound-healing complications in patients with colon neoplasms. However, the monoclonal antibody showed no significant difference for breast cancer, metastatic renal cell carcinoma, non-small-cell lung cancer and gastro-oesophageal adenocarcinoma (Figure 4).

**Figure 5:**
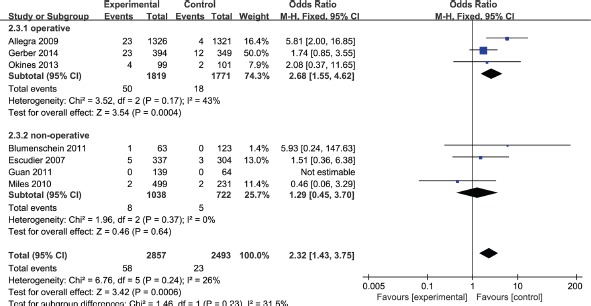
Subgroup analyses by operative status

In previous study, the wound healing problems were defined as abdominal incisional hernia or infusion port dehiscence/inflammation [13]. In terms of the definition, wound might be considered as operative wound in most cases. Therefore, the subgroup analyses stratified by operative status was also required. The result was shown in Figure 5. We have obtained that bevacizumab might be increased risk of wound-healing complications in operative patients.

As a rule of thumb, tests for funnel plot asymmetry should be used only when there are at least 10 studies included in the meta-analysis, because when there are fewer studies the power of the tests is too low to distinguish chance from real asymmetry. In this study, only 7 studies were included in the quantitative meta-analysis. It is hard to rule out the existence of publication bias by visual inspection of the funnel plot, and we therefore did not evaluate publication bias.

## DISCUSSION

This systematic review and meta-analysis is a more comprehensive update that systematically and quantitatively evaluates the relationship between bevacizumab and wound-healing complications in patients with various oncological indications. The results revealed that bevacizumab might be increased risk of wound-healing complications in patients with a variety of tumors. Furthermore, the finding was consistent in subgroup analyses for colon cancer. However, for the breast cancer, metastatic renal cell carcinoma, non-small-cell lung cancer and gastro-oesophageal adenocarcinoma, bevacizumab didn't show the effects of increased risk of wound-healing complications.

As of now, only one systematic review on the topic has been published [17]. In the systematic review, 8 studies from 2005 to 2011 including 7 observational studies and 1 RCT were enrolled. However, observational studies are potentially subject to selection bias [18], and should be interpreted cautiously. Thus, this review limited the analysis to randomized controlled trial which are the gold standard of clinical research [19], to ensure that only the highest quality data were used. Besides, several RCTs were conducted after 2011. A systematic review and meta-analysis need to reflect current research. Updating reviews is necessary when new studies are found. Consequently, we performed a meta-analysis to assess the effect of bevacizumab on wound-healing complications in cancer patients.

With bevacizumab expands in clinical use in the oncological setting [20], awareness of its specific toxicity profile will become increasingly important, especially for the plastic surgeon who will increasingly be entrusted with proper wound care and elective reconstructions in these patients [21]. Our meta-analysis demonstrated that bevacizumab might be increased risk of wound-healing complications in cancer patients especially for colon cancer. However, the specific timing, and nature of bevacizumab-induced wound-healing complications did not emerge in this study. These features are increasingly crucial to guide therapy and outline optimal evidence-based management recommendations. In terms of current literature, bevacizumab should occur at least 60 days before or 28 days after surgery [8], should not be initiated until all wounds are fully healed, and should be permanently discontinued for wound dehiscence [22]. In addition, bevacizumab should also be suspend before elective surgery, though the interval was remain controversial, with some recommending 4 weeks [23] and others 6 to 8 weeks [24]. Nevertheless, the recommendation of the interval largely rely on preclinical pharmacokinetics evidence, as bevacizumab's long circulating half-life of 20 days [25], [26].

A major strength of this meta-analysis was the compliance with the PRISMA guidelines and the recommendations of the Cochrane Collaboration. In order to increase the robustness of this meta-analysis, we enrolled only high-quality and adequately powered RCTs. Several potential limitations should be taken into consideration when interpreting the present results. First of all, the included studies in our meta-analysis were conducted in various oncological indications patients. Thus, the risk of introducing potentially significant heterogeneity is imminent. In addition, patient variables including age, gender, underlying disease, and nutritional status were also the potential bias factor [27, 28]. Other limitations of this study included that the sample sizes were not large and unpublished studies were not included in the analysis. These factors may have resulted in bias.

In conclusion, the present systematic review and meta-analysis suggests that bevacizumab increase the incidence of wound-healing complications for cancers especially for perioperative colon neoplasms patients. However, the adverse effect is not appeared in breast cancer, metastatic renal cell carcinoma, non-small-cell lung cancer and gastro-oesophageal adenocarcinoma. Unfortunately, the findings rely chiefly on data from single or two studies. Thus, the current clinical evidence is not of high enough quality to guide clinical application. Further research is required to assess the wound-healing complications risk of bevacizumab in each oncological indication. In addition, bevacizumab-surgery interval correlates with WHC risk is also required to evaluate.

## MATERIALS AND METHODS

### Selection criteria

Studies meeting the following criteria were included: (1) population: patients with oncological diagnoses receiving bevacizumab; (2) intervention: bevacizumab with or without concurrent chemotherapy; (3) comparison: chemotherapy or no agent; (4) outcome: the incidence of wound-healing complications; (5) design: randomized controlled trials (RCTs).

### Search strategy

Pubmed, Embase and the Cochrane Library, were searched to identify RCTs that referred to the wound-healing complications of bevacizumab in various solid tumors. All the data were searched from inception of the database to June 2016. The following search terms were used: ‘wound healing’, ‘wound’, ‘complication’, ‘bevacizumab’, ‘avastin’, ‘cancer’, ‘tumor’, ‘carcinoma’ and ‘neoplasms’. The search strategy is shown in Table 2. No language restriction was imposed. The reference lists of all retrieved articles were also reviewed to identify additional articles missed by using these search terms.

**Table 2:** Search strategy

Search Terms	
#1	bevacizumab OR avastin
#2	wound healing OR wound OR complication OR adverse reactions
#3	cancer OR tumor OR carcinoma OR neoplasms
#4	#1 AND #2 AND #3

### Selection of studies and data extraction

Two investigators (Zhang and Huang) independently carried out the initial search, deleted duplicate records, screened the titles and abstracts for relevance, and identified each as excluded or requiring further assessment. We reviewed the full-text articles designated for inclusion and manually checked the references of the retrieved articles and previous reviews to identify additional eligible studies. Discrepancies were resolved by consensus.

### Risk of bias assessment

Two reviewers (Zhang and Huang) independently evaluated the methodological quality of identified studies. The ‘risk of bias tool’ referred to the Cochrane Handbook for Systematic Reviews of Interventions version 5.1.0 was used to assess methodological quality [29]. In terms of the assessment criteria, each study was rated and assigned to one of the three following risk of bias: low: if all quality criteria were adequately met, the study was deemed to have a low risk of bias; unclear: if one or more of the quality criteria was only partially met or was unclear, the study was deemed to have a moderate risk of bias; or high: if one or more of the criteria were not met, or not included, the study was deemed to have a high risk of bias [30].

### Statistical method

Data were analyzed using the Review Manager 5.1.0. statistical package (Cochrane Collaboration Software). Dichotomous outcomes were expressed as odds ratio (OR) with 95% confidence intervals (CI) [31]. Heterogeneity among the included studies was evaluated by the *I*² test. A value greater than 50% to indicate substantial heterogeneity and sought the potential sources of heterogeneity (clinical heterogeneity and methodological heterogeneity) [31]. If the results of the studies could not combine using meta-analysis (due to significant clinical heterogeneity and unconventional methods used in the analysis of studies), they were just only presented individually.
